# Why it Worked: Participants’ Insights into an mHealth Antiretroviral Therapy Adherence Intervention in China

**DOI:** 10.2174/1874613601812010020

**Published:** 2018-03-12

**Authors:** Lora L. Sabin, Lauren Mansfield, Mary Bachman DeSilva, Taryn Vian, Zhong Li, Xie Wubin, Allen L. Gifford, Yiyao Barnoon, Christopher J. Gill

**Affiliations:** 1 Department of Global Health, Boston University School of Public Health, 801 Massachusetts Avenue, Crosstown, 3rd floor, Boston, MA, 02118, USA; 2 University of New England, 716 Stevens Ave, Portland, ME, 04103, USA; 3 FHI 360, Room B110, Floor 4, Building 1, No.15, Guanghua Road, Chaoyang District, Beijing, 100026, China; 4 Department of Global Health, Milken Institute School of Public Health, George Washington University, 2121 I St NW, Washington, D.C., 20052, USA; 5 Department of Health Policy and Management, Boston University School of Public Health, 715 Albany Street, Talbot Building, T348W, Boston, MA, 02118, USA; 6 Center for Healthcare Organization and Implementation Research, VA Boston Healthcare System, 150 S. Huntington Ave, Boston, MA, 02130, USA; 7 Boston Children’s Hospital, 300 Longwood Ave, Boston, MA, 02115, USA

**Keywords:** HIV treatment, ART adherence, Behavior change, Intervention trial, mHealth, China

## Abstract

**Background:**

Few Antiretroviral Therapy (ART) adherence trials investigate the reasons for intervention success or failure among HIV-positive individuals.

**Objectives:**

To conduct qualitative research to explore the reasons for effectiveness of a 6-month mHealth (mobile health) trial that improved adherence among ART patients in China. The intervention utilized Wireless Pill Containers (WPCs) to provide, real-time SMS reminders, WPC-generated adherence reports, and report-informed counseling.

**Methods:**

We conducted in-depth interviews with 20 intervention-arm participants immediately following the trial. Sampling was purposeful to ensure inclusion of participants with varied adherence histories. Questions covered adherence barriers and facilitators, and intervention experiences. We analyzed data in nVivo using a thematic approach.

**Results:**

Of participants, 14 (70%) were male; 7 (35%) had used injectable drugs. Pre-intervention, 11 were optimal adherers and 9 were suboptimal adherers, using a 95% threshold. In the final intervention month, all but 3 (85%) attained optimal adherence. Participants identified a range of adherence barriers and facilitators, and described various mechanisms for intervention success. Optimal adherers at baseline were motivated by positive adherence reports at monthly clinic visits-similar to receiving A+ grades. For suboptimal adherers, reminders facilitated the establishment of adherence-promoting routines; data-guided counseling helped identify strategies to overcome specific barriers.

**Conclusion:**

Different behavioral mechanisms appear to explain the success of an mHealth adherence intervention among patients with varying adherence histories. Positive reinforcement was effective for optimal adherers, while struggling patients benefitted from reminders and data-informed counseling. These findings are relevant for the design and scalability of mHealth interventions and warrant further investigation.

## 1 INTRODUCTION

Interventions to improve adherence to Antiretroviral Therapy (ART) medication regimens among people living with HIV (PLHIV) are critical to achieve optimal outcomes. Poor adherence has serious consequences for patients, including progression of HIV to AIDS, development of resistant strains of HIV, and death [1-7]. ‘Optimal adherence’ is often interpreted as ≥95%, though other thresholds have been used [6, 8, 9], particularly in recent years as the potency of current ART regimens suggests that lower adherence may be associated with viral suppression [10]. However, sustaining high ART adherence is a challenge for many patients, with typical ART adherence ranging between 60-80% [6, 11-14] and often declining over time [4, 15-18]. A number of interventions have been shown to improve ART adherence [19-22]. Successful intervention approaches have included elements such as providing information to patients, engaging them in discussions of cognition and adherence expectations, directly-observed therapy, nutrition support, and drug-use treatment [22]. Mobile health (mHealth) interventions that use real-time monitoring strategies for ART are a new approach that allow early identification of adherence lapses [23-25]. mHealth adherence interventions have been shown to be effective in resource-limited settings, and among certain populations, such as people who inject drugs (PWID), that often face specific challenges to achieving optimal adherence [25-27].

Relatively few studies have investigated the factors that determine success or failure of adherence interventions, including those focused on ART. Candy *et al*., reviewed randomized trials using qualitative evidence on patients’ views to help understand variation in the effectiveness of interventions to improve adherence to long-term drug therapy [28]. Their analysis identified common intervention components; the component most commonly associated with effective interventions was ‘a focus on personal risk factors’. Ineffective interventions typically lacked this component. Other components of successful interventions included ‘explaining the value of adherence’ or ‘provision of clear/appropriate information on how to take medication’ [28]. While these findings are useful for informing the design of future interventions, they do not fully explain the mechanisms behind the behavioral change(s) that result in improved adherence or maintenance of optimal adherence at the individual level.

The China Adherence Through Technology Study (CATS) assessed efficacy of a novel mHealth adherence intervention among HIV-infected individuals in China [29]. China is home to Asia’s second largest HIV epidemic, with over 500,000 individuals living with HIV and indications that the annual rate of new infections is rising [30]. China’s western and southern border regions have been hardest hit by HIV, largely due to easy access to and high use of heroin in these areas [31]. Although China’s government has scaled up ART rapidly, with nearly 300,000 individuals initiated on ART by 2015 [30], drug resistance is emerging as a major concern, a sign of suboptimal adherence [32, 33]. The CATS trial was one of the first rigorous trials to assess an adherence intervention in China, and the first to report on the efficacy of an intervention based on new real-time adherence monitoring technology [29]. One component of the trial was to explore the views and experiences of trial participants regarding the intervention, particularly regarding use of the Wireless Pill Containers (WPCs) that monitor adherence in real-time. In the present analysis, we examined the results of this qualitative component. It encompassed In-Depth Interviews (IDIs) with a sample of CATS trial participants to better understand the dynamics of intervention results. Specifically, we aimed to explore the mechanisms of action behind success of the intervention, and whether they varied according to adherence at baseline.

## 2 MATERIALS AND METHODS

### 2.1 CATS Study Site and Intervention

The CATS study was conducted from December 2012 to April 2014 in Nanning, China, capital city of Guangxi Province, and home to 7 million residents. Located on China’s southern border north of Vietnam, Guangxi has experienced a widespread heroin epidemic that has helped fuel the spread of HIV: recent estimates suggest that 80,000-100,000 individuals had been infected with HIV by 2011 [34]. We implemented CATS at the ART clinic operated by the Guangxi Provincial Center for Disease Control and Prevention (CDC). In 2012, the clinic was staffed with 4 physicians, 2 nurses, and 3 HIV counselors, and provided HIV treatment to over 1,000 patients. Most clinic patients were on a twice-daily ART regimen consisting of nevirapine or efavirenz, plus lamivudine with stavudine or lamivudine with zidovudine; patients initiating therapy typically started on a once-daily regimen of lopinavir/ritonavir plus tenofovir or abacavir. Usual care involved monthly clinic visits for medication refills and frequent sessions with adherence counselors at the request of a clinician or patient.

For the CATS trial, we enrolled 120 ART patients and provided each one with a WPC for one ART medication, with the medication selected by the participant based on pill fit and patient preference for refilling frequency. The WPC (Wisepill Technologies, Capetown, South Africa) recorded the date and time of each container opening and communicated the data immediately to a central server. After three months of passive monitoring, participants were stratified by “optimal” *vs* “suboptimal” adherence, based on a 95% threshold over the full 3 months. We then randomized participants within each stratum to intervention or comparison arms for the 6-month trial. Intervention participants received personalized text message reminders triggered when the WPC was not opened within 30 minutes past prescribed dose time. They also received a WPC-generated report showing their adherence in the previous month at each monthly clinic visit. Participants with suboptimal adherence in the prior month were required to participate in counseling provided by trained adherence counselors using the reports; optimal adherers in the previous month had a choice of engaging in report-informed counseling. The trial’s methods are described in detail elsewhere [29].

The CATS intervention resulted in improved mean on-time ART adherence and a greater likelihood of achieving ‘optimal adherence’, as defined by 95% on-time adherence [29]. The results showed an immediate and sustained improvement in mean adherence among intervention participants who were suboptimal adherers (defined prior to implementing the trial as <95% at baseline). Among optimal adherers at baseline, mean adherence remained above 95% for the full intervention period (6 months) for intervention participants, whereas it declined among comparison participants [29].

### 2.2 Qualitative Study Participants

Participants in the IDIs were all CATS trial subjects, and thus had met study criteria at the time of enrollment. They were: receiving or initiating ART; aged 18 years or above; owned a mobile phone; and deemed at risk for poor adherence by clinicians or themselves. Trial participants’ time on ART was an average of 2.5 years at randomization [29]. For the post-intervention qualitative component, we purposefully identified 20 intervention arm subjects to maximize a range of experiences (roughly 1/3 of intervention arm participants). This sample size was pre-determined based on the team’s previous experience with the number of participants needed to reach saturation. First, we selected all seven self-reported PWID. Next, we identified subjects randomly to yield equal numbers of optimal vs. suboptimal adherers at baseline. Finally, we made adjustments to ensure inclusion of 3-4 female participants in each group (equal to their overall proportion among trial subjects).

### 2.3 Data Collection

All 20 pre-selected subjects agreed to participate in an IDI at their sixth and final intervention visit to the study clinic (Month 9 of the study). A trained interviewer, who had also served as the coordinator of the CATS trial, conducted each IDI in Mandarin Chinese, using a semi-structured interview guide. The interview guide provided open-ended questions with follow-up probes on perceived adherence barriers and facilitators, and participants’ experiences participating in, and views of, the CATS intervention. These encompassed features such as use of the WPC, reminders, adherence reports, and data-informed counseling. Typical questions were: “Can you tell me about your experience with the text message reminders?”, “Can you tell me about your feelings when you saw your adherence report?”, “Can you tell me about your experiences with the counseling?”. The average length of the IDIs was 30 minutes (ranging from 20-50 minutes); all were audio-recorded. Demographic information was collected at enrollment into the CATS study.

The study was reviewed and approved by the institutional review boards at Boston University Medical Center in Boston, Massachusetts, USA and the Guangxi Provincial CDC in Nanning, China. All participants provided written informed consent.

### 2.4 Data Analysis

The IDI audio-recordings were transcribed by one team member verbatim and translated into English by two dual Chinese-English speakers. The resulting Chinese language and English language transcripts were then reviewed by a third dual language-speaker for accuracy. Four Boston-based team members (two of whom were dual-language speakers) read all the transcripts and analyzed the English-language versions in QSR NVivo 11. A preliminary analysis by two members yielded major themes. Two additional team members started with these themes and used an iterative approach to further explore them and sub-themes in depth. For instance, an initial theme was “positive views of the counseling.” As further reading was pursued, we noted that participants commented on the ways in which counselors helped them to surmount adherence barriers. We thus created a sub-theme, “help from counselors in overcoming adherence obstacles.” In this way, a comprehensive theme codebook was developed to code each transcript systematically. We compared responses by baseline adherence, adherence at month 9 (final intervention month), gender, and PWID status. Responses were prioritized by their frequency; we also examined divergent views.

## 3 RESULTS

### 3.1 Baseline Characteristics of Study Participants

Of all participants, 14 (70%) were male (Table **1**). Seven (35%) were PWID and seven also reported using non-injectable drugs (35%); six (30%) reported using both types of drugs. Three (17.65%) said they drank alcohol at least 3-4 times per week. The majority (15, 75%) were employed. Nine participants (45%) had been on ART for less than 6 months. At randomization, 11 (55%) were optimal adherers and nine (45%) were suboptimal adherers. In the final intervention month, 10 (90.91%) of the 11 baseline optimal adherers and seven (78%) of the nine baseline suboptimal adherers attained ≥95% adherence. The three who failed to achieve optimal adherence were: 1) a young male who was suboptimal at baseline; 2) a middle-aged male who was optimal at baseline; and 3) a middle-aged female who was suboptimal at baseline. None were among those with self-reported drug use. Individual-level information on key parameters for each participant is provided in Table **2**. Fig. (**1**) contains illustrative reports for optimal and suboptimal adherers at two time points: Month 3 (final month before randomization) and Month 5 (second month of the intervention).

Our analysis revealed several themes related to participants’ perceptions of adherence and their perceived barriers and facilitators. Clear themes and sub-themes also emerged with respect to the intervention. These findings are presented below, beginning with views toward adherence generally, and then covering barriers and facilitators, and finally, views of the intervention. Direct statements are presented where appropriate. Where differences emerged by baseline adherence, adherence in the last intervention month, gender, and drug use, these are highlighted.

### 3.2 Views Regarding Adherence

All participants said that being adherent was of personal importance. This stemmed from their understanding of the clinical necessity of ART and that being adherent would keep the disease under control. Statements about adherence were intermingled with a sense of confidence and optimism, even among suboptimal adherers. As one such participant explained: “*I am confident in taking my medicine continuously.”* (Male, 28, baseline suboptimal)

Most participants stated that they felt personally responsible for their health, and that the rationale for adhering to therapy was to ensure effective treatment and good health:

*It’s [taking the medication] for myself. I want to live a better life and to be healthier.* –Female, 29, baseline suboptimal

*Taking my medicine is the top priority and nothing could change our punctuality in taking medicine, no matter how busy we are.* - Male, 40, baseline optimal

### 3.3 Perceived Barriers and Facilitators

Several themes emerged related to perceived barriers and facilitators of adherence. The main barriers mentioned were job-related issues, feared stigma related to HIV, and unplanned events. Facilitators encompassed having a daily routine, using reminders, and social support. These are each discussed below.

#### 3.3.1 Barriers

Work-related barriers were noted by most (75%) participants. Most typically, they explained that they were busy at work and would either forget to take their medication or would be unable to *“get away”* to take a dose. As one participant, a truck driver, explained:

*The biggest challenge is that, when I’m driving on the highway, *etc*., there is no rest stop. For example, I could take my medicine from 10 to 12, but I may have already been on the highway before 10 and still haven’t reached the next service stop at 12. In that case, I’d miss the time for my medication.* –Male, 33, baseline suboptimal.

Certain types of employment, namely those that allowed for breaks, were viewed as more supportive of medication-taking than others. For example:

*What I did for a living before [hairdresser], I can’t just stop what I was doing and take my medicine immediately…. It will also be a problem for waiters in restaurants. But for cashiers working in a supermarket or internet-café, they may just excuse themselves by saying that they need to drink some water*. –Male, 28, baseline suboptimal

Divergent views emerged regarding which types of employment posed barriers. In contrast to the statement above, a female participant who worked as a shelf-stocker noted that being a cashier could be challenging for timely dose-taking: “*Cashiers cannot pause [to take medication].”* (Female, 33, baseline suboptimal)

Another barrier was fear of revealing HIV status, both within and beyond work settings. Many participants said they wanted to hide their status from their boss or coworkers, making it imperative to find a private place to take their medications. This could be challenging, as explained by two participants:

*Once I was on a business trip and I was half an hour late taking my medicine. That’s because the bag with my bottle in it was stored in the trunk of the car and my boss was driving. The car didn’t stop then, and I was too shy to ask him to pull over.* -Male, 35, baseline optimal

*Sometimes I have meetings until very late and I couldn’t just walk away. It was a bit inconvenient in those situations…. We have strict rules for meetings. We were not allowed to leave freely…. We were not even allowed to take phone calls. -*Male, 35, baseline optimal

Fear of inadvertent disclosure was also an issue for those whose social network (*i.e.*, family, friends) remained unaware of their status. Most participants’ families and/or partners knew their status, but some did not. This could lead to dose-taking delays. As two participants with suboptimal adherence explained:

*I was having a walk with another person, and there was no water [to take the medication with] along the way. I couldn’t just leave…. There was no bathroom either. Nothing.* -Female, 30, baseline suboptimal

*It feels really troublesome. I’m worried that other people will see me opening the pill bottle and wonder what medicine I have in there. They will feel strange…. I was worried about my friends.* -Female, 29, baseline suboptimal

Another major theme was the importance of having a routine, and conversely, the way that unexpected events jolted routines, requiring negotiation and often resulting in late or missed doses. In these cases, participants could not plan ahead to ensure timely dose-taking:

*There are some unexpected situations. Our lives are not very stable, nor are our jobs. I’m basically a freelancer, so sometimes things just happen out of the blue. Everything is possible.* -Male, 40, baseline optimal

In addition, one participant, a PWID, highlighted the effect of drinking alcohol. In his words: “*Sometimes I forgot. I drank too much and got drunk.”* (Male, 35, baseline suboptimal). Another PWID acknowledged the negative consequences of illegal drug use, noting that he had been compelled to take a dose late when hiding from the police.

#### 3.3.2 Facilitators

Regarding adherence facilitators, most participants stressed the importance of maintaining or establishing a routine as the key to sustaining optimal adherence. Having a routine meant incorporating dose-taking into daily activities on a regular basis:

*It feels like a part of my life and when it’s time, I’d take them. I have been on medication for so long, I won’t forget at all. When it’s time, I definitely will get them out and take them, just like having meals every day.* -Male, 42, baseline optimal.

This theme was consistent among all groups of participants, with failure to establish and maintain a routine emerging as a reason for lack of improved adherence during the intervention. In the words of one suboptimal adherer at baseline as he reflected on his persistently low adherence: *“The main problem was a lack of routine.” (Male, 22, baseline suboptimal)*

Another facilitator was the presence of a reminder system. Many participants reported relying on an alarm-most frequently a phone-based reminder-to prompt them to take a dose. As one participant explained:

*Usually when my alarm goes off, I’d grab the bottle out of my bag with one hand and snooze the alarm with my other hand. Therefore, I don’t think I could miss my medicine.* -Male, 35, baseline optimal

A third facilitator was having social support. Participants stressed that it helped them to be with people who knew their HIV status, and described the role that their families and friends played in reminding them to take their doses. Several participants referenced their *“patient friends”*, referring to HIV-positive friends. They alluded to social activities, as well as conversations about the WPC, with these friends:

*People in my circle basically know about it. I have a friend now. He checks my text messages …. and then he’d tell me to be on time next time.* -Female, 35, baseline suboptimal

*My family knows about it, [and] so do my patient friends. Except for my patient friends, I was worried about being seen [with the WPC] by other people.* -Male, 40, baseline optimal

All participants also reported a good relationship with the clinicians at the study clinic; most described feeling supported by the doctors and counselors, including all of the PWID. Their descriptions revealed their high regard for the counselors, whom they claimed conveyed *“mutual understanding.”* One female PWID explained:

*Sometimes when we have something bothering us in our life, they would also comfort me. It feels like having a friend at the same level. I’m not that suppressed like when I’m outside.* -Female, 38, baseline optimal.

Only one participant suggested that encounters with a clinician might be unsupportive. This participant, a male whose adherence was suboptimal at baseline and failed to improve over the intervention, said that if he did not take his medications as directed, *“I would be criticized.”* (Male, 22, baseline suboptimal)

Several specifically mentioned that the counselors played a supportive role regarding ART. This is an illustrative statement:

*I think it also serves as mental assistance [talking to clinicians or counselors]. It is possible that nobody around us knows that we are taking this medicine…. Only when I’m here for the consultation, would I tell the consultants my difficulties. -*Male, 20, baseline optimal

### 3.4 Effect of the Intervention

Five major themes emerged that were related directly to the effect of the intervention, including: salutary “supervisory effect;” the motivating influence of objective feedback; the role of the reports in promoting accountability; text messages as simple reminders or to establish routines; and the usefulness of counseling in overcoming barriers.

#### 3.4.1 Salutary “Supervisory Effect”

Participants described a beneficial supervisory effect of the intervention, particularly WPC use. The specific words they used included *“self-imposed pressure,” “invisible pressure,” “a supervisor,”* and *“like a teacher overseeing.”* Participants were aware that the WPC sent wireless signals that recorded their adherence, and they liked the sense of ‘being watched.’ In this sense, the WPC also served as a cue to action, urging participants to take their medication. Participants also described how the WPC helped condition them to establish a habit of timely dose-taking:

*I think the bottle is equivalent to an invisible pressure to remind myself of taking medicine on time. It seems I won’t forget when I see it. Now, I basically begin to think that it’s half an hour for me to take my medicine at 9:30, and whatever I’m doing, I will have this in mind.* -Male, 20, baseline optimal

Most of the time, participants revealed positive feelings about this “supervisory effect,” claiming that it was a helpful type of pressure that supported high adherence. Only one male participant, suboptimal at baseline, expressed an opposing view, saying that *“the self-imposed pressure was ever growing,”* causing him to worry frequently. (Male, 22, baseline suboptimal)

#### 3.4.2 Objective Feedback *via* Reports led to Greater Awareness and Motivation

All participants liked the adherence reports, claiming that they were a helpful reference for maintaining or improving adherence. Participants said the reports provided a visual depiction of their punctuality that was easy to read and to use for identifying late or missed doses. The reports heightened their awareness of recent medication-taking behavior, including changes in such behavior:

*Now I see 96.6 [percent] for this month and it used to be over 98. I would certainly be aware of the wrong time taking my medicine in this month. I can’t make the same mistake the next month. It just reminds me to have this awareness.* -Female, 38, baseline optimal

*I was not punctual at the beginning…. It’s better when I have that …[the adherence report] as reference. You can know about your situation through this…. I can read it and if I was not punctual, I’d pay more attention.* -Male, 28, baseline suboptimal

Participants described feeling happy and satisfied when they received a report showing optimal adherence in the previous month. Participants who were optimally adherent at baseline stressed the way that the report was encouraging and reinforced positive adherence behaviors. As one male participant explained: “*When I see the straight line…. I’d feel satisfied for the past month. It makes me happy and I think it’s encouraging.”* (Male 26, baseline optimal) Participants who had late or missed doses reported feeling badly. However, this was generally viewed as motivation to improve the report for the next month. One participant explained:

*It is [helpful]. The time before last time, I missed once and I felt pretty bad about it. Once I was late and only took it after the set time. I quite care about this 100% [goal].* -Male, 33, baseline suboptimal

#### 3.4.3 Reports Promoted Accountability

While personal motivation was important, many participants revealed a desire to please the clinicians, to *“follow the doctor’s orders”* (Male 42, baseline optimal). One participant explained his motivation for being adherent: “*Half for my own health, and half … to do what the doctors want me to do.”* (Male, 40, baseline optimal)

Participants viewed their relationship with the doctor as somewhat contractual and being adherent as a *“promise”* to the doctors. They saw themselves as trustworthy and did not want to break this promise. One participant explained:

*I care about other people’s opinions when I do something and I wish to do it well…. I take my promise seriously and I’m a man of my word. When I break my promise, the first thing I do is to blame myself, and next, I’d be concerned about whether other people are angry and think that I’m not trustworthy.* -Male, 33, baseline suboptimal

To this end, the report provided an objective record of adherence that promoted a sense of accountability to doctors, counselors, and to the patients themselves.

*The pillbox can prove that I have been taking my doses on time, and the clinicians would know that I am adherent…. to let them know I have been taking my doses on time, and I am following their instruction.* - Male, 32, baseline optimal

*For example, [in the past] it would be natural to tell them that I didn’t miss my medication when they ask. [Now] had I really missed, even if I could lie about it, I’d still feel hesitant and I’d feel perturbed.* -Male, 26, baseline optimal

*As for the [adherence report], he [the counselor] said to me, “look, you didn’t take your medicine on time, it looks like a wave”. I said, “really? I thought I was on time”…. Did you notice that I have more straight lines now?* -Male, 28, baseline suboptimal

#### 3.4.4 Text Messages as Simple Reminders or to Establish Routines

Overall, participants viewed the text messages as a useful reminder if a dose had been forgotten. Occasionally signals were not transmitted immediately upon opening of the WPCs, so some participants reported receiving a reminder after having taken their medication, though this was not viewed as burdensome. A typical statement about the reminders was: “...*definitely useful. For example, it would remind you if you forget to take your medicine. The feeling is that I’d think about whether I had forgotten to take my medicine. I’d recall whether I had forgotten and then take it if I had*.” (Male, 35, baseline suboptimal)

Views of the text messages differed by adherence at baseline. Most optimal adherers said that they would have been adherent without the reminders. However, they also noted that in the case of an unplanned event, unusual business, or when a dose was forgotten, the text messages were helpful:

*I think they [reminders] are useful as I can double check whether I have had my medicine. I can take it then, if I haven’t.* -Male, 35, baseline optimal

*I have my alarm set at 9:45 and 9:55 separately. Sometimes I was busy doing homework or other things when the alarm went off. I wanted to wait to take my medicine right on time, so I just kept on doing what I was doing until it was 10:30, when the message came in. Then I’d realize that I forgot to take my medicine, and I’d take it immediately.* -Male, 20, baseline optimal

Some high adherers noted that the text messages were a general reminder of the need for consistent dose-taking:

*This pill bottle is a reminder. You have to remember to take your medicine on time; otherwise you’ll get a text message. That’s how I feel. I think it helps you to form the idea that you have to take your medicine when it’s time*. -Female, 38, baseline optimal

Among suboptimal adherers at baseline, most participants viewed the text messages as helpful both in alerting them to missed doses and in helping them to establish a routine to support on-time adherence behaviors. Only a few said they thought the reminders were not critical because they would still use an alarm. A typical statement was:

*I remember that this thing would remind me later; I would just hurry and take it [medication]. Otherwise, I’d get a text message reminder later. Because of this mindset, I must hurry to open the bottle and take my medicine within half an hour. That’s the type of mindset that pushes me to hurry, to be comparatively more punctual.* -Female, 29, baseline suboptimal

One participant said that she had used an alarm previously, but had come to rely on the text messages instead because they were more discreet.

*I used to set an alarm, but I don’t set it very often now.... Don’t you think that people might wonder why you always run away when it was time? Now I set it on vibration. …I already remember the time now after a while…. For now, I’ll just rely on this text message.* -Female, 33, baseline suboptimal

#### 3.4.5 Usefulness of Counseling in Overcoming Barriers

Among participants who engaged in data-informed counseling, which was required only when the report showed <95% adherence in the prior month, all were positive about the counseling. This is an illustrative statement about the counseling during the intervention, by the same participant who noted that counselors could sometimes be critical:

*I [learned] more about adherence. I could forget if I was told just once, but through repeated counseling, I would take the information more seriously. The counselors gave me different examples, like a patient who could always take his ARVs [antiretroviral medications] on time or a patient who was not adherent, and the consequences.* -Male, 22, baseline suboptimal

Over the course of the intervention, many participants identified a personal barrier to adherence and then devised a strategy to overcome it. Sometimes these were simple strategies like finding excuses to leave to go take their medication when out with friends or at work. Sometimes it was a matter of planning ahead how to take a pill more discreetly or punctually.

*At the beginning, I used a small bag and I couldn’t open my pill bottle in the open public. My bag then was narrow and I could only open it vertically…. Now I have a bigger bag, I can open it horizontally.* -Male, 28, baseline suboptimal

*I think it takes some planning ahead…. I need to have the pill bottle with me all the time. You have to carry your bottle when you’re not sure whether you can get back in time.* - Male, 35, baseline optimal

*I’d take it in bed when I’m in my dorm because there are lots of things in my bed, which could serve as a screen. Plus my dorm mates wouldn’t notice when they are playing games. When I’m out, using the bathroom is the most frequently used excuse, as this is normal.* -Male, 20, baseline optimal

Many participants enacted a strategy suggested to them by a counselor. As two participants explained, including one who remained suboptimal at the end of the intervention:

*For instance, [they suggested that I] change my habit and set the alarm clock ahead of my dose time. Now, I don’t go to bed even if I am very tired and sleepy. I would wait until I take my doses.* -Male, 22, baseline suboptimal

*The pill bottle makes some noises when it’s getting empty. They told me to put some cotton wool in it.* -Male, 20, baseline optimal Occasionally, counselors advised not using the WPC in order to facilitate dose-taking. As the participant quoted above explained: *“[Counselors also said that] if I were truly afraid of being seen by other people, I could take the meds out in advance and put them somewhere easy to reach, which simplifies the process.”* (Male, 20, baseline optimal)

While most participants reported success in overcoming personal barriers to achieve optimal adherence, one participant who failed to achieve optimal adherence explained his inability to change certain habits:

*I have changed my habit of going to sleep too early. I am limiting my computer game playing as well. I would try to set one more alarm [on my cellphone] 10 minutes after the first alarm, but I feel that would also be a problem. It is a bit annoying. When I play computer games, I cannot stop*. -Male, 22, baseline suboptimal

## 4 DISCUSSION

This qualitative study aimed at exploring the reasons that an mHealth ART adherence intervention trial in China proved successful in helping HIV-positive participants who were suboptimal adherers to improve their adherence, and in supporting maintenance of high adherence among those who began the trial with optimal adherence. Generally, we found that perceptions of the value of adherence, as well as perceived facilitators and barriers played an important role in adherence behaviors for participants. All participants showed a clear understanding of the importance of adherence and motivation to achieve optimal adherence. That they understood that adherence was key to successful treatment may reflect the fact that the study took place in a large city in China that had been providing ART services for some time, with the result that patients had absorbed the messaging provided by clinicians and counselors regarding adherence. Most also revealed strong internal motivation to achieve optimal adherence. Thus, lack of understanding or motivation to adhere does not explain poor adherence among those participants who failed to maintain or achieve optimal adherence by the end of the trial.

Participants described a number of issues they believed acted as barriers to adherence. These included: work issues, fear of revealing HIV status, and unplanned events. Both employment-related barriers and stigma have been well documented in the ART adherence literature, including in China [35-40]. Unplanned events-or changes in one’s regular schedule-has also been a common ART adherence barrier in numerous settings [39, 40], along with the related phenomenon-forgetfulness [39, 41]. One previous study has shown a link between a ‘chaotic life’ (self-reported) and missed outpatient visits among HIV-positive individuals [42]. Missed outpatient visits are a likely indicator of poor ART retention and thus may also be indicative of poor adherence since retention in care is required for adherence to a medication regimen.

In our analysis of these qualitative data, five clear themes emerged that help clarify the reasons for the trial’s positive effect on adherence. These encompassed: a beneficial “supervisory effect” of the WPC; the motivating effect of receiving objective adherence feedback; a sense of accountability that was encouraged by adherence reports; the way that triggered reminders served as simple reminders and/or helped establish routines; and the role of counseling in overcoming barriers. Woven through these themes was a strong recognition of the importance of personal awareness and accountability; these were consistent across adherence level at baseline, gender, and prior drug use. In part, this recognition may be due to the fact that the majority of participants had been on ART for some time even before trial enrollment-they were thus already experienced (and in some sense ‘successful’ since they were retained in care) patients. Yet the intervention itself seemed to heighten such recognition in several important respects.

First, the “supervisory effect” of the WPC was viewed as beneficial. Participants liked being “watched,” claiming that it naturally increased personal awareness of adherence. While our patients expressed this positive view, not all patients will welcome a potential “Big Brother” feeling [43]. Second, the monthly adherence reports emerged as key to enlightening participants about their monthly adherence patterns. This finding is less controversial; a recent review and meta-analysis of adherence-enhancing interventions in 79 RCTs, including an assessment of “drug dosing histories” (like the adherence reports used in CATS), found that giving patients feedback about their dosing behavior was the largest factor influencing adherence [44]. Finally, the reports were instrumental in providing an objective record of the prior month’s adherence that motivated participants to be accountable to themselves and to the doctors. The desire to appear trustworthy and not disappoint one’s doctor may be a form of introjected motivation (actions taken to avoid guilt or shame). Other studies have suggested that striving to save face may motivate behavior change in some populations [45, 46].

While we found no clear differences in experiences or views by gender or PWID status, the differences between those who were baseline optimal vs. baseline suboptimal adherers represent a key finding of this study. For participants who already had high adherence, the intervention clearly provided support and motivation to maintain high adherence. In contrast to those who were suboptimal at baseline, optimal adherers had already established positive habits and a routine of on-time adherence prior to intervention implementation. For this group, the reminders served mainly as a reminder in rare cases of forgetfulness. However, the adherence report played an important role for this group. They viewed the report as something akin to a report card. The receipt of a report that showed declining adherence, or even worse, suboptimal adherence in the previous month, was viewed as a warning sign. This finding may help to explain why the CATS intervention arm did not show the typical decline in adherence over time observed in other studies. While declines in adherence have been commonly observed in prior studies, usually they are characterized as a “modest decline” in adherence [15-18]. Although the threshold of 95% adherence is arbitrary, and a slight decline below 95% may not have serious medical consequences, reports showing a decline to below 95% clearly prompted participants to redouble their efforts to cross back over the 95% threshold. They desired a ‘good report,’ and wished to avoid the sense of failure they associated with a ‘suboptimal’ report.

For participants who were baseline suboptimal adherers, the intervention seems to have worked through two key mechanisms, regardless of gender or experience with drug use. First, the triggered text messages played an important role in helping participants to establish habits that would lead to a pattern of on-time adherence. This is important because the establishment or maintenance of a routine was fundamental to achieving optimal adherence over the course of the intervention. The direct link between behavior (being late for a dose or headed toward missing a dose altogether) and receiving a reminder may have helped provide an immediate stimulus for behavior change. Here, participants’ knowledge of the importance of adherence, and their motivation to be adherent, are critical, encouraging them to view the triggered reminders as a welcome support, rather than as annoying messages unassociated with behavior, as automatic reminders have been viewed in other studies [47-52].

Second, the WPC-generated adherence reports and data-informed counseling were critical in helping suboptimal participants overcome whatever personal barriers they were experiencing. As our findings show, the data-informed counseling in particular presented an opportunity to discuss barriers and to strategize about solutions. Here, the positive relationship with clinicians that participants described is particularly relevant. Were they to fear criticism, or were clinicians unprepared for a supportive conversation, the counseling might not have contributed to the positive effect found in CATS. Interestingly, the evidence on the potential impact of data-informed counseling is growing, including from some of our previous work in China [9, 44, 53]. However, this study is the first of which we are aware that provides clear evidence from ART patients themselves about how data-informed counseling assisted them in overcoming barriers. Further studies to unpack the details of what is shared in these types of counseling sessions may provide guidance about how to best use data to inform counseling.

Notably, few participants failed to achieve optimal adherence over the course of the intervention-only three among the qualitative study sample. Little emerged from the qualitative data to help shed light on why the intervention did not succeed with these participants. Indeed, the themes and sub-themes that surfaced from the data were generally consistent among all participants regardless of whether their adherence was optimal in Month 9. The most notable exception was the view expressed by one participant, a young male whose adherence remained suboptimal throughout the intervention, who felt that being watched led to an “ever growing” self-imposed pressure that made him anxious. This particular participant also conveyed somewhat mixed experiences with counseling: he stated that he would be ‘criticized’ when he was not adherent, and yet also conveyed positive views of the data-informed counseling, giving examples of how the counselors helped him cope with specific pill-taking challenges. It would thus appear to be the case that while struggling participants liked the intervention, and most of its separate features, it did not *always* spur optimal medication-taking behaviors.

These findings have several important implications for informing the design of mHealth interventions for HIV adherence, as well as for the scalability of this type of intervention. Our results regarding the central role of personal barriers are notable because it suggests that what participants had previously lacked were strategies and support to overcome these barriers. They support those of Candy *et al.*, who found that a focus on overcoming personal risk factors was a common thread of successful interventions for adherence [28].

In addition, our findings suggest that for ART patients who are already high adherers, real-time monitoring combined with reports may provide sufficient support. However, further counseling appears necessary to help suboptimal adherers overcome challenges. Our findings also help to address concerns as to whether the use of triggered text-message reminders could train patients to take their medications only in response to a reminder, which might leave patients vulnerable in the event of device failures or loss of cellular connectivity. The CATS study found that the proportion of doses taken before the 30-minute mark increased during the intervention period, mitigating this concern [29]. The qualitative component corroborates this finding, indicating that text message reminders served to help participants establish a habit of on-time adherence such that they no longer relied on the messages as medication reminders.

### 4.1 Limitations

We note several study limitations. First, the topics discussed in the IDIs-views of HIV and adherence, motivations, values, accountability, and relationship with clinicians, are all highly culture-specific and findings related to these factors will undoubtedly vary in different settings and populations. Second, we were limited by the fact that the majority of our participants succeeded in attaining optimal adherence by month 9, restricting perceptions of adherence ‘failure’. Similarly, we used a binary (yes/no) measure to determine baseline adherence (suboptimal versus optimal), as well as intervention success, defined as ≥95% adherence in month 9. This may have limited our comparisons between suboptimal and optimal adherers at baseline, as such dichotomous categories do not capture the full extent of variability within these categories. In addition, although electronic drug monitors are considered the gold standard in adherence measures, we could not determine the reliability of the WPCs we used in CATS. It is possible that participants opened the WPC because they knew they were being monitored, but did not take their medication. However, we believe a more significant limitation may be that participants would occasionally take pills out of the container in advance for later dosing without the device recording that they took the dose. Several participants reported doing this when they could anticipate situations during which it would be difficult to use the device. Unfortunately, the HIV viral load outcomes from the trial are not helpful in understanding the effect of this activity, primarily because most participants were suppressed at randomization [29]. Finally, participants may have provided biased information due to poor recall or out of a desire to please the interviewer, a common challenge in qualitative research. In this case, we do not believe the latter was an issue because the interviewer was also the study coordinator at the study clinic and was familiar with the issues that participants faced with their medications, having given them their detailed adherence reports and discussed challenges with them over the previous 6 months of the intervention.

## CONCLUSION

This study provides valuable insight regarding the factors driving the success of an mHealth intervention to promote HIV adherence. Our findings help to elucidate the role of the different components of the CATS intervention in contributing to change in adherence behaviors resulting in improvement or maintenance of optimal adherence. Notably, our findings show the differential effect of the intervention observed among participants who were baseline optimal adherers compared with baseline suboptimal adherers. These findings warrant further investigation because of their implications for the design and scalability of mHealth interventions. Interventions should give consideration to patients’ prior level of adherence, and should ensure that patients are adequately supported to overcome personal barriers to adherence, if necessary.

## Figures and Tables

**Fig. (1):**
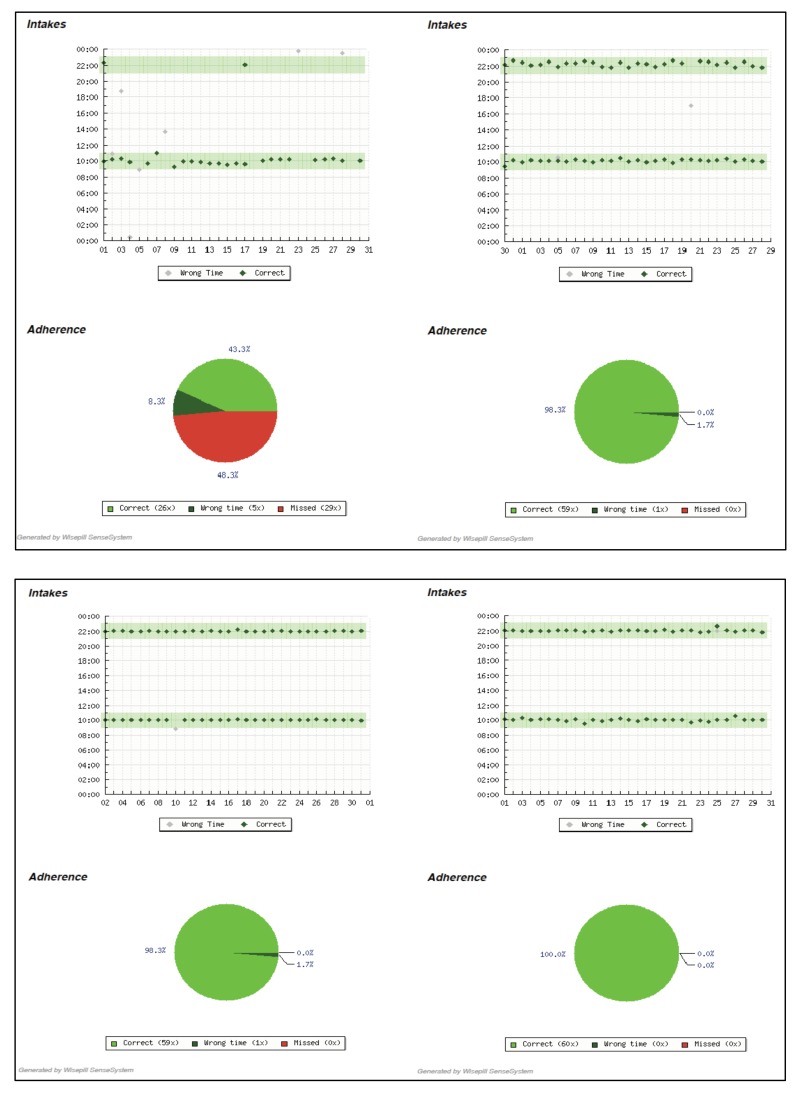
Examples of Wireless Pill Container adherence reports. Graphs show the adherence history in the pre-intervention period (Month 3, in graphs to the left) and in the second intervention month (Month 5, in graphs to the right) for two subjects. 2a (top): Reports for a patient with suboptimal adherence; 2b (bottom): Reports for a patient with optimal adherence.

**Table 1:** Characteristics of participants at baseline.

**Demographic Characteristics**	Number (%/SD)
Age, years, n (%) 18-24	2 (10.0)
25-34	10 (50.0)
35-44	8 (40.0)
Male, n (%)	14 (70.0)
Highest education level achieved, n (%)	
Primary school only	2 (10.0)
Middle/secondary school	12 (60.0)
Beyond secondary school	6 (30.0)
Married, n (%)	7 (35.0)
Employed, n (%)	15 (75.0)
Monthly income, yuan, mean (SD)	2678.5 (2197.7)
**Health Characteristics**	
Optimal adherence ≥95%, n (%)	11 (55.0)
Time on ART, months, mean (SD)	23.8 (28.4)
Time on ART <6 months, n (%)	9 (45.0)
Twice/daily (vs. once/daily) regimen, n (%)	11 (55.0)
Used injectable street drug (ever), n (%)	7 (35.0)
Used non-injectable drug (ever), n (%)	7 (35.0)
Used both injectable and non-injectable drug (ever), n (%)	6 (30.0)
Alcohol use 3-4 times per week or greater, n (%)	3 (17.7)

**Note:** Most characteristics were similar to those of the entire intervention-arm sample in the CATS trial, with the exception of injectable and non-injectable drug use, which was 11% and 13%, respectively [29]

**Table 2:** Key Characteristics of Individual Participants.

**Participant Number**	**Age (at time of IDI)**	**Gender**	**Self-Reported Injectable Drug use (yes/no)^1^**	**Self-Reported Non-Injectable Drug use (yes/no)^2^**	**Pre-Intervention Adherence** **(Months 1-3)^3^**	**Adherence in Last Intervention Month (Month 9)^3^**
1	32	M	Yes	Yes	optimal	optimal
2	22	M	No	No	suboptimal	suboptimal
3	20	M	No	No	optimal	optimal
4	40	M	Yes	Yes	optimal	optimal
5	35	M	Yes	No	suboptimal	optimal
6	38	F	Yes	Yes	optimal	optimal
7	28	M	No	Yes	suboptimal	optimal
8	26	M	No	No	optimal	optimal
9	34	M	Yes	Yes	optimal	optimal
10	30	F	No	No	suboptimal	optimal
11	33	F	No	No	suboptimal	optimal
12	29	F	No	No	suboptimal	optimal
13	35	M	No	No	optimal	suboptimal
14	40	M	Yes	Yes	optimal	optimal
15	33	M	No	No	suboptimal	optimal
16	32	M	No	No	suboptimal	optimal
17	35	F	No	No	suboptimal	suboptimal
18	42	M	Yes	Yes	optimal	optimal
19	35	M	No	No	optimal	optimal
20	31	F	No	No	optimal	optimal

^1^Yes denotes heroin use; ^2^of 7 who self-reported non-injectable drug use: 6 had smoked heroin, 2 had taken methadone, 2 had snorted or swallowed ketamine, 1 had snorted or swalled amphetamines or methamphetamines, and 1 had smoked opium; ^3^optimal adherence refers to ≥ 95% adherence and suboptimal adherence refers to <95% adherence as measured by a real-time wireless pill monitor.
